# The Role of Peer Patient Navigation in Enhancing PrEP Persistence Among LGBTQ + Populations and Other Individuals at High Susceptibility to HIV Transmission in the Deep South State of Alabama

**DOI:** 10.1007/s10461-025-04911-8

**Published:** 2025-10-22

**Authors:** Karen A. Johnson, Chuong Bui, Heather K. Graham, Christa Mayfield, Pamela Payne-Foster, David L. Albright, D. Scott Batey

**Affiliations:** 1 The University of Georgia School of Social Work, Athens, GA USA; 2 The University of Alabama, Alabama Life Research Institute, Tuscaloosa, AL USA; 3 The University of Alabama College of Education, Counselor Education, Tuscaloosa, AL USA; 4 Birmingham AIDS Outreach, Magic City Wellness Center, Birmingham, AL USA; 5 The University of Alabama at Birmingham Heersink School of Medicine, Department of Bioinformatics and Data Science, Birmingham, AL USA; 6 The University of Alabama College of Arts and Sciences, Tuscaloosa, AL USA; 7 Tulane University School of Social Work, New Orleans, LA USA

**Keywords:** PrEP, LGBTQ+, Deep south, Peer patient navigation

## Abstract

This study evaluated the impact of peer patient navigation compared to standard care in promoting PrEP treatment among individuals at high risk for HIV in Alabama. The study was conducted at an LGBTQ+-focused primary care clinic dedicated to culturally competent, affirming care. Longitudinal data from January 2016 to June 2022, covering 4,194 visits by 764 patients, were analyzed. Three service periods were compared: Standard of Care (SOC) (01/2016–05/2018), PrEP-Up! (06/2018–06/2021), and Enhanced PrEP-Up! (07/2021–06/2022). Segmentation regression was employed to analyze (1) the effects of PrEP-Up! and Enhanced PrEP-Up! on appointment persistence (versus “standard of care”); and (2) the effect of COVID-19. Participants were 71.5% White, 19.2% Black, 3.5% Other race, and 5.8% Unknown race. Regarding ethnicity, 10.6% disclosed as Not Hispanic/Latino, 3.6% as Hispanic/Latino, 2% as Central American/Chicano, and 84.9% had ethnicity unavailable. 93.5% identified as male, with 56.9% identifying as LGBTQ+. Persistence rates under SOC averaged 88%. PrEP-Up! improved persistence to 95% initially but declined slightly over time. COVID-19 significantly disrupted persistence during early 2020, though rates gradually improved with the easing of restrictions. During the Enhanced PrEP-Up! period, persistence increased more slowly, possibly reflecting residual pandemic effects. Peer patient navigation effectively enhanced PrEP persistence in a region disproportionately impacted by HIV. Despite challenges posed by COVID-19, these findings emphasize the importance of community-centered interventions in addressing health disparities. Rebuilding trust in public health among marginalized populations is essential to improving equitable health outcomes.

## Introduction

Despite significant advances in HIV prevention efforts over the past four decades, LGBTQ + populations continue to face disproportionately high rates of new transmission, with Black or African American men (“Black” hereafter) who have sex with men (MSM) experiencing the highest rates [1]. Rates of new transmission among other highly burdened populations such as Black trans women are also disturbingly high [1, 2]. In Deep South states like Alabama, new transmission rates among highly burdened LGBTQ + populations are even higher in rural regions as compared to non-rural locations [3–5]. This has been attributed in part to HIV stigma and prejudice related to sexual orientation. These barriers are often felt more intensely in rural parts of the highly conservative Deep South states [6, 7]. Stigma and prejudice have—at least in part—contributed to the existing barrier of distrust of healthcare providers for persons with HIV (PWH) [8]. Unsurprisingly, three of the seven states recently identified as priority national Ending the HIV Epidemic (EHE) jurisdictions due to having at least 10% of new diagnoses in rural areas were in the Deep South (Alabama, Mississippi, and South Carolina) [9]. 

Among key EHE strategies promoted is the promotion of pre-exposure prophylaxis (PrEP) to improve HIV prevention [10]. This preventive approach has proven highly effective, demonstrating a 99% success rate in preventing HIV transmission through sexual encounters and at least 74% effectiveness in averting transmission through injection drug use [11]. Despite its clear effectiveness, however, PrEP remains largely underutilized among LGBTQ + populations, with even lower rates of usage among those facing greater social and structural vulnerability in the Deep South such as Black MSM and Black trans women [6, 12]. 

Recent findings revealed that 66% of PWH interviewed in a Southern hospital were not aware of PrEP, with the majority indicating that they would have encouraged their partners to use it had they been aware [13]. In a recent Alabama study, while Black MSM accounted for 50% of new HIV diagnoses, this population represented only 18% of those accessing PrEP services [14]. Although far less studied, significant disparities in PrEP awareness and usage have also been identified among Black trans women residing in this region of the US [15]. While a lack of PrEP uptake (i.e., initiation) is cause for concern, persistence—that is, maintained PrEP usage as prescribed—also presents a challenge [16]. Limited evidence exists regarding how to best promote and implement PrEP usage in Deep South communities with high HIV transmission risk. This represents a serious health equity gap. This gap is particularly significant given the increased stigma related to HIV and bias directed at LGBTQ + populations in the Deep South. Alabama has some of the lowest rates of PrEP utilization and the highest rates of new HIV transmissions among Black MSM in the nation [6, 17, 18]. 

One method of encouraging PrEP uptake and maintenance in highly burdened populations is peer patient navigation (PPN) [19]. Adapted from its original use with cancer patients, PPN connects vulnerable persons to healthcare and promotes preventative and treatment services; beyond those points, patient navigators vary in their definitions [19]. One study found several common areas addressed by PPN programs, including health education, linkage to care, care coordination, transportation support, and other forms of related assistance [20]. PPN is also noted to be of particular benefit to persons who encounter individual, interpersonal, social, and structural barriers to accessing care, such as those leaving prison and re-entering the community [21]. A lack of evidence exists, however, regarding the effectiveness of peer patient navigators in encouraging PrEP prevention and treatment engagement among LGBTQ + populations residing in the Deep South. In fact, to date, no studies exist that have examined the use of PPN to promote PrEP usage among MSM living in this region of the US. This is despite evidence confirming PPN’s effectiveness in addressing social and structural risks like those that are highly concentrated in the Deep South such as lack of transportation access, and its proven success in promoting engagement in HIV care and sustaining viral suppression among men living with HIV [20, 22, 23]. Additional barriers to HIV prevention and treatment engagement as a whole include setting and/or provider mistrust, medication mistrust, perceived racism, and stigma [24, 25]. 

This manuscript addresses this gap in literature. In it, we utilize retrospective longitudinal service utilization data collected from a healthcare facility in Alabama to examine monthly PrEP-related clinic visits among predominantly MSM and other LGBTQ + populations using longitudinal data spanning from 01/04/2016 to 06/01/2022, encompassing 4194 clinic visits by 764 patients. Three successive time periods of PrEP treatment services were included in the analysis. During the latter two treatment periods, PrEP was administered using two different levels of PPN services titled, respectively, PrEP-Up! and Enhanced PrEP-Up! For these latter two periods, we examined the effects of PPN services on appointment persistence versus standard of care (SOC). The effect of COVID-19 was also examined given the time period examined.

### Primary Care Setting

The study site is a primary care setting and the first and only LGBTQ + comprehensive healthcare facility in Alabama. Located in an urban center, the primary care setting is committed to providing culturally competent care and a safe, open, and affirming space for LGBTQ + individuals residing across the urban-rural continuum. PrEP care has been provided at the clinic since 2016. In addition to PrEP, services include primary care, hormone replacement therapy, and testing and treatment for sexually transmitted infections (STIs).

## Methods

### IRB Approval

Approval to conduct the analysis was obtained from the Institutional Review Board at The University of Alabama (IRB Protocol ID: 22-11-6141).

### Descriptions of Study Observation Periods

The three distinct observation periods are described below.

*Period 1 Establishing a PrEP SOC (Standard of Care)*. The SOC period began when the primary care setting opened its doors on 01/04/2016, and lasted through 05/31/2018. Almost immediately, the primary care setting began offering PrEP care as a standard clinic service. PrEP care, in the form of tenofovir disoproxil fumarate + emtricitabine taken in a single pill (brand name Truvada) daily for HIV prevention, was offered to individuals at high likelihood of HIV transmission, following the March 2014 US Public Health Service comprehensive clinical practice guidelines for PrEP [26]. During this period, PrEP risk(s) assessments were conducted by providers during routine medical visits and with identified clinic primary care patients likely to benefit from PrEP or through self-referral in which an individual specifically requested PrEP access. Over the 28-month SOC period, 471 individuals began receiving PrEP at the primary care setting.

*Period 2 PrEP-Up!* The PrEP-Up! period lasted from 06/01/2018–06/30/2021. Following the development of SOC PrEP services and monitoring of subsequent PrEP uptake at the clinic, the primary care setting was awarded funds on June 1, 2018. These funds came from the Alabama Department of Public Health (ADPH)/Centers for Disease Control and Prevention (CDC) to address health disparities related to PrEP uptake and persistence. Specific populations prioritized in PrEP-Up! were minoritized MSM, heterosexuals, and high-risk negative (HRN) individuals in the Birmingham-Hoover (Alabama) Metropolitan Statistical Area (MSA). PrEP-Up! aimed to reflect both the current local HIV epidemiologic landscape and existing community efforts to address gaps along the HIV Care Continuum.

Integrated into the primary care setting, SOC, and provision of both FTC/TDF (Truvada) and FTC/TAF (Descovy), PrEP-Up! provided individual- and structural-level means to improve PrEP knowledge, uptake, and persistence through: (1) Personalized Cognitive Counseling (PCC), a CDC evidence-based intervention to reduce high-risk behaviors among MSM who are repeat HIV testers; (2) Retention through Enhanced Personal Contacts (REPC), a CDC evidence-based intervention adapted by the organization for PrEP linkage and persistence. This intervention includes routine enhanced reminder calls (6–8 days and 3 days before appointments), enhanced follow-up calls within 24–48 h of missed visits, and assessment of barriers to appointment attendance; (3) HIV counseling, testing, and linkage to care; and (4) broad structural-level marketing of PrEP, associated PrEP care, and PrEP access. PrEP-Up! added two full-time staffers, both licensed social workers, devoted to achieving programmatic goals [27, 28]. During these 37 months of service, the number of individuals prescribed PrEP at the primary care setting increased by approximately 83% over the SOC period.

*Period 3 Enhanced PrEP Care*. Enhanced PrEP Care was delivered from 07/01/2021 and continues to this day (though the observation period itself ended on December 31, 2022). Enhanced PrEP Care was characterized by enhanced contact with current and potential PrEP users. Specifically, a peer patient navigator was hired whose primary responsibilities were to conduct Enhanced PrEP Care activities. The peer patient navigator brought essential qualities to PrEP persistence efforts, including lived experience within the local community and an established role as a trusted community gatekeeper. These characteristics fostered a sense of trust which strengthened patient engagement and encouraged adherence to PrEP care. In addition to routine meetings with the peer patient navigator prior to and/or after their PrEP appointment, a key component of this period was the scheduling of the next appointment approximately 90 days out. These changes were influenced by the introduction of updated PrEP care guidelines from the CDC that included care for PrEP through cabotegravir (CAB) injections. The COVID-19 pandemic, the resultant social isolation orders, and subsequent changes in personnel practices also led to an increase in virtual contact with patients during this period. The primary care setting director also initiated at least monthly data queries of clinic PrEP services that were ultimately presented to the medical team, the PrEP services peer patient navigator, the program evaluator, and the agency Board of Directors. During the 17-month window of examination, Enhanced PrEP Care resulted in an approximate 26% increase in those prescribed PrEP at the primary care setting over the PrEP-Up! period.

### Statistical Analysis

Descriptive statistics were used to categorize the sociodemographic characteristics of the study population.

The analysis included SOC dates of service that began 1.5 years after the organization started providing PrEP services to the community. June 1, 2018, was selected to ensure that the window of analysis captured a period when the organization had reached a steady state of operation in its provision of PrEP services. In a given month, if a patient had another visit within the following six months, the patient’s persistence was coded as Yes (1). Otherwise, persistence was coded No (0). The length of the persistence window was determined by the PrEP-Up! evaluation team and was timed in view of the multiple dosing options for PrEP, with the goal of staying connected to the patient.

Persistence was assessed for 3,922 visits from 663 patients that took place between 06/01/2018 and 06/30/2022. Table 1 was excerpted from the data for illustration. For instance, Patient A had a visit on 11/15/2017. They had the next visit on 8/30/2018, which is not within six months of 11/15/2017; persistence was coded No (0). Patient B had a visit on 11/30/2017. They had the next visit on 3/06/2018, which is within six months of 11/30/2017; persistence was coded Yes (1).

**Table 1:** Illustration of appointment persistence assessment

Client ID	Service date	6 months from service date	Next visit	Persistence
A	11/15/17	5/15/18	8/30/18	0
B	11/30/17	5/30/18	3/06/18	1
C	11/10/20	5/10/21	3/18/21	1
D	10/28/19	4/28/20	5/27/20	0
E	5/23/19	11/23/19	.	N/A
F	5/16/22	11/16/22	.	N/A

Various reasons exist that a patient may stop coming for PrEP appointments. Typically, those reasons are not disclosed. In this study, reasons for persistence (or non-persistence) are not assessed. For each patient, persistence was not assessed for the last recorded visit. The following examples explain the rationale. Patient E had a last recorded visit on 5/23/19. There may have been a variety of reasons that the patient stopped coming for service (e.g., relocation, no longer at risk for HIV, etc.). Because this patient stopped coming for PrEP, persistence was, therefore, not assessed. Another case in point is Patient F who had a visit on 5/16/22. It was unclear (from the available data that stretched to 12/31/22) whether this patient stopped coming or had a visit in 2023. Persistence was, therefore, not assessed.

**Fig. 1:**
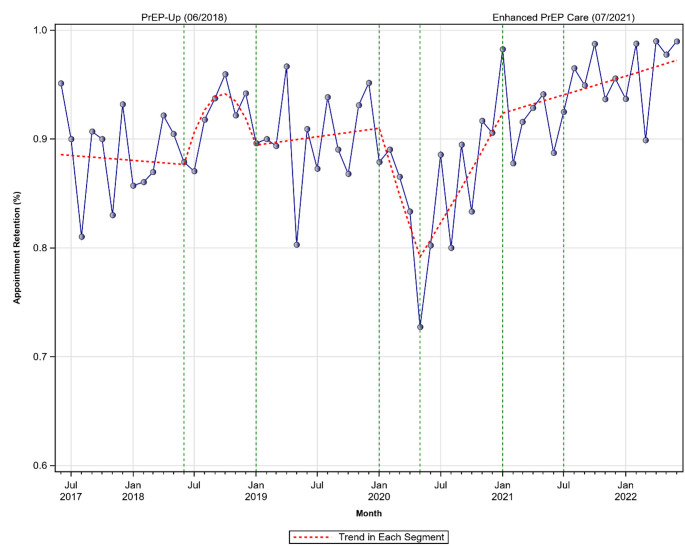
Appointment Persistence, Monthly Data from 6/2017 to 6/2022. The trends in the periods 6/2017-6/2018 and 1/2019-1/2020 are not flat because the estimated slopes are not exactly zero (see Table 3)

The time periods under analysis were reflective of SOC (06/01/2017-05/31/2018), PrEP-Up! (06/01/2018-06/30/2021), and Enhanced PrEP Care (07/01/2021-06/30/2022). Counts of persistence = 1 was generated for each month, resulting in clinic-level monthly data of appointment persistence from 06/2017 to 06/2022 (Fig. 1). Piecewise regression was used for the analysis to examine if PrEP-Up! resulted in significant changes in levels and/or trends of appointment persistence, as compared to the SOC period. A total of six regression splines were used to capture different trends in the six different segments of the monthly data of appointment persistence (Fig. 1). Details of how S_1_-S_6_ were created are provided in the appendix.

Monthly counts of persistence = 1 was the dependent variable. S_1_-S_6_ were the independent variables of interest. In order to capture the ∧-shaped trend from 06/2018 to 01/2019, the regression model included a quadratic term S_2_squared_. Included as covariates were % of White patients, % of patients with commercial insurance, and % of male patients. These variables were aggregated at clinic-level for each month. The regression model was estimated using Poisson regression with the logarithm of the number of visits (in each month) as the offset variable. Standard errors are adjusted by Pearson’s chi-square divided by degrees of freedom to account for overdispersion. PROC GENMOD in SAS/STAT 15.1 was used for model estimation [29]. 

## Results

As demonstrated in Table 2, patient characteristics included 71.5% White, 19.2% Black, and 3.5% Other race. The vast majority were Male (93.5%); most patients were covered by commercial insurance (88.7%). Latinx individuals accounted for at least 5.6% of the sample, but ethnicity was not available for the majority of patients (84.9%). Regarding sexual orientation, 56.9% identified as LGBTQ+, 3.5% as heterosexual, 0.8% as other, and 38.9% did not disclose. The vast majority of participants were from urban counties in Alabama (86%), but there was representation from rural counties in Alabama (10.4%) and out-of-state (3.6%) as well.

**Table 2:** Patient characteristics

Characteristic	Category	Percentage (%)
Race	White	71.5
	Black	19.2
	Other	3.5
Gender	Male	93.5
	Female	6.5
Insurance coverage	Commercial	88.7
Ethnicity	Latinx	5.6
	Ethnicity not available	84.9
Sexual orientation	LGBTQ+	56.9
	Heterosexual	3.5
	Other	0.8
	Did not disclose	38.9
Geographic distribution	Urban counties in Alabama	86.0
	Rural counties in Alabama	10.4
	Out-of-state	3.6

Table 3 presents results from the piecewise regression examining the effects of PrEP-Up! As reflected in Table 3; Fig. 1, persistence fluctuated around 88% between 06/2017 and 06/2018 without a significant trend (S_1_, b = − 0.001, *p* =.748). For several months after the start of PrEP-Up!, persistence exhibited a ∧-shaped trend (S_2_, b = 0.037, *p* =.027; S_2_squared_, b = − 0.005, *p* =.031), rising from about 88% to 95% between 06/2018 and 10/2018 and then decreased to about 90% between 10/2018 and 01/2019. Persistence fluctuated around 90% without a significant trend for the year of 2019 (S_3_, b = 0.002, *p* =.420). Results suggested that PrEP-Up! helped increase appointment persistence from 88% to 90%, as compared to SOC.

**Table 3:** Piecewise regression examining effects of PrEP-Up!

	b	se		*p*
S_1_	−0.001	0.003	0.10	0.748
S_2_	0.037	0.017	4.87	0.027
S_2_squared_	−0.005	0.002	4.67	0.031
S_3_	0.002	0.003	0.65	0.420
S_4_	−0.035	0.008	18.28	<0.001
S_5_	0.019	0.004	22.38	<0.001
S_6_	0.003	0.002	4.00	0.045
%White	0.055	0.102	0.29	0.590
%Commercial insurance	−0.212	0.164	1.68	0.195
%Male	0.025	0.204	0.02	0.903

S_1_: estimated trend from 6/2017-6/2018; S_2_: 6/2018-1/2019; S_3_ : 1/2019-1/2020; S_4_ : 1/2020-5/2020; S_5_ : 5/2020-1/2021; S_6_ : 1/2021-6/2022

%White = % Caucasian/White patients; %Male = % of male patients; %Commercial insurance = % of patients with commercial insurance

As also reflected in Table 3, persistence showed a sharp drop during the first half of 2020 (S_4_, b = − 0.035, *p* <.001), reaching a record low of about 75% in 05/2020. The decline was followed by an increase during the second half of 2020 (S_5_, b = 0.019, *p* <.001), bringing persistence to a high of 98% in 01/2021. Persistence continued to trend upward between 01/2021 and 06/2022 but at a lower rate than the preceding period (S_6_ = 0.003, *p* =.045).

## Discussion

This study addresses a significant gap in peer-reviewed literature related to the use of peer patient navigators in delivering enhanced PrEP services as compared to SOC in promoting PrEP care to LGBTQ + populations and other individuals at high susceptibility to HIV transmission. We examined service-level retrospective data to examine three successive time periods of PrEP treatment services. Results suggest that incorporating peer patient navigators, who bring lived experiences and cultural resonance and are trained to deliver evidence-based interventions, can positively influence engagement with PrEP treatment. PPN-specific features of the PrEP-Up! phase included (1) the introduction of PCC to reduce HIV transmission behaviors; (2) the utilization of REPC, a system of personalized and enhanced reminder calls for appointments and assessment and mitigation of barriers to care, as a way to improve appointment adherence; and (3) the broad structural-level marketing of PrEP and its associated care services. The program added two full-time licensed social workers to support these efforts. Enhanced PrEP introduced additional enhancements to patient engagement through the hiring of a peer patient navigator whose primary responsibility was to conduct Enhanced PrEP Care activities. This period emphasized routine in-person and virtual (due to COVID-19 restrictions) meetings with patients before and after appointments and focused on scheduling future appointments 90 days in advance. To our knowledge, this is the first study examining PPN promotion for PrEP persistence in the US Deep South, and findings point to the effectiveness of PPN services delivered in this region of the US in at least two important ways.

First, the transition from the SOC period to the PrEP-Up! period was associated with a significant increase in persistence levels. This is despite the well-documented challenges associated with PrEP utilization in the US Deep South among LGBTQ + populations broadly, and Black MSM and Black trans women in particular. Additional mixed methods research is needed to understand the reasons for such sharp increases in engagement. However, the peer-based strategies utilized in PrEP-Up! may have mitigated barriers that previously hindered PrEP uptake. Given the available evidence, these barriers may have included mistrust of organizational settings, providers and/or medication, perceived racism, and/or stigma [24, 25]. These results are consistent with prior research indicating the potential of peer-based interventions to improve health outcomes among populations made vulnerable by intersecting social and structural risks [30]. By establishing a connection between peers and healthcare providers, individuals may have felt more comfortable seeking care and discussing their concerns openly. As prior research makes clear, peer patient navigators can serve as HIV-related health advocates, reducing the stigma associated with HIV prevention services and creating an environment where sexual minority individuals feel supported and understood [31]. 

Second, the Enhanced PrEP-Up! period provides an additional perspective on the impact of PPN, particularly within the context of the COVID-19 pandemic. Findings that persistence rebounded after the lifting of pandemic restrictions suggest that PPN may have played a critical role. While it remains unclear to what extent the presence of PPN contributed to this rebounding, existing research suggests a synergistic potential between PPN and other professional supports, though more research is needed to determine the scope of its potential impact [32, 33]. Not only did peer patient navigators support the recovery of persistence rates to pre-pandemic levels, but they also contributed to a marginal increase over previous PrEP care time periods, an outcome that is particularly notable given the ongoing challenges of pandemic-related isolation.

The adaptability of PPN—such as leveraging virtual communication and follow-up strategies—may have mitigated a sharper decline in persistence that would have otherwise been expected during this time. Their efforts likely helped sustain patient engagement and adherence to PrEP treatment despite the challenging circumstances. PPN reminders and follow-up calls were not location-specific and could be provided over the phone. This eliminated the need for face-to-face time between patients and navigators, demonstrating the adaptability of this approach. This flexibility aligned with the goals of the Enhanced PrEP-Up! program, which aimed to foster a positive relationship between patients and providers and to provide personalized counseling and persistence strategies. The Enhanced PrEP-Up! phase demonstrates how PPN can play a unique role in fostering resilience in healthcare delivery systems during public health crises. To date, this is the first study published that evaluates the contributions of PPN within broader systemic challenges, such as the COVID-19 pandemic, to better understand their capacity to address barriers to care during times of social and structural adversity.

## Limitations

While this study offers valuable insights to efforts to end the HIV epidemic, there are limitations to consider. Firstly, the retrospective nature of the data and the focus on a single clinic in Alabama limit the generalizability of the findings to other settings. Future research could explore the scalability of PPN programs in various regions, considering cultural and geographical differences. Secondly, persistence exhibited a sharp decline in the beginning of 2020. While we attribute this decline to the COVID-19 epidemic, reasons for persistence or non-persistence were not assessed. Another significant limitation of this study is the potential underrepresentation of non-persistence. Individuals who ceased attending PrEP care appointments were not categorized as non-persistent because such cases could potentially be due to circumstance (e.g., moving out of town). This approach may inadvertently exclude cases of true non-persistence, creating a potential gap in measuring comprehensive adherence and follow-up. Lastly, this study did not investigate potential variations in PrEP uptake and persistence among racial and/or ethnic groups within LGBTQ + populations. Given the significant disparities in HIV transmission rates among different racial and ethnic groups, this limitation constrains understanding of differences within and across different racial and ethnic identities.

## Conclusion

This study is among the first to examine the role of PPN, as compared to SOC, in promoting PrEP treatment to LGBTQ + populations and other individuals who have a high burden of HIV transmission in the US Deep South. Results highlight the effectiveness of PPN in bridging PrEP gaps in this highly conservative region of the nation. Amidst ongoing efforts to end the HIV epidemic, the incorporation of peer patient navigators into healthcare settings in states like Alabama may enhance the delivery of PrEP and other HIV prevention treatments while mitigating social and structural barriers such as stigma and mistrust.

## Appendix

Variable *Time* indicates time in months between 6/2017 and 6/2022, running from 0 to 60. Variable *Time* was used to create the regression splines S_1_-S_6_ as follows. Supplemental Table 1 was provided for illustration.

$$\:{S}_{1}=\left\{\begin{array}{c}time,\:\:\:\mathrm{f}\mathrm{o}\mathrm{r}\:time\:\le\:12\\\:12,\:\:\mathrm{o}\mathrm{t}\mathrm{h}\mathrm{e}\mathrm{r}\mathrm{w}\mathrm{i}\mathrm{s}\mathrm{e}\end{array}\right.$$

$$\:{S}_{2}=\left\{\begin{array}{c}\begin{array}{c}0,\:\:\mathrm{f}\mathrm{o}\mathrm{r}\:time\:\le\:12\:\:\\\:time-12,\:\:\:\mathrm{f}\mathrm{o}\mathrm{r}\:12\le\:time\:\le\:19\end{array}\\\:7,\:\:\mathrm{o}\mathrm{t}\mathrm{h}\mathrm{e}\mathrm{r}\mathrm{w}\mathrm{i}\mathrm{s}\mathrm{e}\end{array}\right.$$

$$\:{S}_{3}=\left\{\begin{array}{c}\begin{array}{c}0,\:\:\mathrm{f}\mathrm{o}\mathrm{r}\:time\:\le\:19\:\:\\\:time-19,\:\:\:\mathrm{f}\mathrm{o}\mathrm{r}\:19\le\:time\:\le\:31\end{array}\\\:12,\:\:\mathrm{o}\mathrm{t}\mathrm{h}\mathrm{e}\mathrm{r}\mathrm{w}\mathrm{i}\mathrm{s}\mathrm{e}\end{array}\right.$$

$$\:{S}_{4}=\left\{\begin{array}{c}\begin{array}{c}0,\:\:\mathrm{f}\mathrm{o}\mathrm{r}\:time\:\le\:31\:\:\\\:time-31,\:\:\:\mathrm{f}\mathrm{o}\mathrm{r}\:31\le\:time\:\le\:35\end{array}\\\:4,\:\:\mathrm{o}\mathrm{t}\mathrm{h}\mathrm{e}\mathrm{r}\mathrm{w}\mathrm{i}\mathrm{s}\mathrm{e}\end{array}\right.$$

$$\:{S}_{5}=\left\{\begin{array}{c}\begin{array}{c}0,\:\:\mathrm{f}\mathrm{o}\mathrm{r}\:time\:\le\:35\:\:\\\:time-35,\:\:\:\mathrm{f}\mathrm{o}\mathrm{r}\:35\le\:time\:\le\:43\end{array}\\\:8,\:\:\mathrm{o}\mathrm{t}\mathrm{h}\mathrm{e}\mathrm{r}\mathrm{w}\mathrm{i}\mathrm{s}\mathrm{e}\end{array}\right.$$

$$\:{S}_{6}=\left\{\begin{array}{c}0,\:\:\mathrm{f}\mathrm{o}\mathrm{r}\:time\:\le\:43\\\:time-43,\:\:\:\mathrm{f}\mathrm{o}\mathrm{r}\:time\:\ge\:43\end{array}\right.$$

**Supplemental Table 1**.

**Table:**

Year	Month	Time	S_1_	S_2_	S_3_	S_4_	S_5_	S_6_
**2017**	**6**	0	0	0	0	0	0	0
2017	7	1	1	0	0	0	0	0
2017	8	2	2	0	0	0	0	0
2017	9	3	3	0	0	0	0	0
2017	10	4	4	0	0	0	0	0
2017	11	5	5	0	0	0	0	0
2017	12	6	6	0	0	0	0	0
2018	1	7	7	0	0	0	0	0
2018	2	8	8	0	0	0	0	0
2018	3	9	9	0	0	0	0	0
2018	4	10	10	0	0	0	0	0
2018	5	11	11	0	0	0	0	0
**2018**	**6**	12	12	0	0	0	0	0
2018	7	13	12	1	0	0	0	0
2018	8	14	12	2	0	0	0	0
2018	9	15	12	3	0	0	0	0
2018	10	16	12	4	0	0	0	0
2018	11	17	12	5	0	0	0	0
2018	12	18	12	6	0	0	0	0
**2019**	**1**	19	12	7	0	0	0	0
2019	2	20	12	7	1	0	0	0
2019	3	21	12	7	2	0	0	0
2019	4	22	12	7	3	0	0	0
2019	5	23	12	7	4	0	0	0
2019	6	24	12	7	5	0	0	0
2019	7	25	12	7	6	0	0	0
2019	8	26	12	7	7	0	0	0
2019	9	27	12	7	8	0	0	0
2019	10	28	12	7	9	0	0	0
2019	11	29	12	7	10	0	0	0
2019	12	30	12	7	11	0	0	0
**2020**	**1**	31	12	7	12	0	0	0
2020	2	32	12	7	12	1	0	0
2020	3	33	12	7	12	2	0	0
2020	4	34	12	7	12	3	0	0
**2020**	**5**	35	12	7	12	4	0	0
2020	6	36	12	7	12	4	1	0
2020	7	37	12	7	12	4	2	0
2020	8	38	12	7	12	4	3	0
2020	9	39	12	7	12	4	4	0
2020	10	40	12	7	12	4	5	0
2020	11	41	12	7	12	4	6	0
2020	12	42	12	7	12	4	7	0
**2021**	**1**	43	12	7	12	4	8	0
2021	2	44	12	7	12	4	8	1
2021	3	45	12	7	12	4	8	2
2021	4	46	12	7	12	4	8	3
2021	5	47	12	7	12	4	8	4
2021	6	48	12	7	12	4	8	5
2021	7	49	12	7	12	4	8	6
2021	8	50	12	7	12	4	8	7
2021	9	51	12	7	12	4	8	8
2021	10	52	12	7	12	4	8	9
2021	11	53	12	7	12	4	8	10
2021	12	54	12	7	12	4	8	11
2022	1	55	12	7	12	4	8	12
2022	2	56	12	7	12	4	8	13
2022	3	57	12	7	12	4	8	14
2022	4	58	12	7	12	4	8	15
2022	5	59	12	7	12	4	8	16
**2022**	**6**	60	12	7	12	4	8	17
